# A predictive model relating daily fluctuations in summer temperatures and mortality rates

**DOI:** 10.1186/1471-2458-7-114

**Published:** 2007-06-19

**Authors:** Anne Fouillet, Grégoire Rey, Eric Jougla, Philippe Frayssinet, Pierre Bessemoulin, Denis Hémon

**Affiliations:** 1 INSERM, U754, Villejuif, France; 2 Université Paris-Sud, IFR69, Epidémiologie Environnementale des Cancers, Villejuif, France; 3 INSERM, CépiDc, Le Vésinet, France; 4 Université Paris-Sud, IFR69, Centre d'Epidémiologie sur les Causes Médicales de Décès, Le Vésinet, France; 5 Météo-France, Toulouse, France

## Abstract

**Background:**

In the context of climate change, an efficient alert system to prevent the risk associated with summer heat is necessary. The authors' objective was to describe the temperature-mortality relationship in France over a 29-year period and to define and validate a combination of temperature factors enabling optimum prediction of the daily fluctuations in summer mortality.

**Methods:**

The study addressed the daily mortality rates of subjects aged over 55 years, in France as a whole, from 1975 to 2003. The daily minimum and maximum temperatures consisted in the average values recorded by 97 meteorological stations. For each day, a cumulative variable for the maximum temperature over the preceding 10 days was defined.

The mortality rate was modelled using a Poisson regression with over-dispersion and a first-order autoregressive structure and with control for long-term and within-summer seasonal trends. The lag effects of temperature were accounted for by including the preceding 5 days. A "backward" method was used to select the most significant climatic variables. The predictive performance of the model was assessed by comparing the observed and predicted daily mortality rates on a validation period (summer 2003), which was distinct from the calibration period (1975–2002) used to estimate the model.

**Results:**

The temperature indicators explained 76% of the total over-dispersion. The greater part of the daily fluctuations in mortality was explained by the interaction between minimum and maximum temperatures, for a day *t *and the day preceding it. The prediction of mortality during extreme events was greatly improved by including the cumulative variables for maximum temperature, in interaction with the maximum temperatures. The correlation between the observed and estimated mortality ratios was 0.88 in the final model.

**Conclusion:**

Although France is a large country with geographic heterogeneity in both mortality and temperatures, a strong correlation between the daily fluctuations in mortality and the temperatures in summer on a national scale was observed. The model provided a satisfactory quantitative prediction of the daily mortality both for the days with usual temperatures and for the days during intense heat episodes. The results may contribute to enhancing the alert system for intense heat waves.

## Background

A weather-mortality relationship in summer and marked excess mortality during extremely hot periods have been clearly established [1,2]. Some studies have described the main epidemiologic and environmental characteristics of specific heat waves [3-14]. Others have focused on a time-series approach in order to model the general temperature-mortality shape or the lag time between a climatic event and its impact on mortality [15-24].

The weather component has mainly been considered on the basis of the temperatures recorded on various lag days. Other climatic parameters, such as humidity, wind speed and pressure, have also been considered as independent variables [17,18,22,24,25] or by constructing combined indices [21] or synoptic patterns [26]. Air pollution has also been included in some studies focusing on specific urban areas [18,20,22,23,25].

Although all the studies have concluded that long and intense heat episodes are responsible for major excess mortality, quantitative indicators that take into account both the intensity and duration of heat episodes have seldom been proposed and formally validated [7,8,21,27,28].

In August 2003, Western Europe experienced a heat wave that was exceptional in terms of its duration, intensity and geographic extent [7,8,27,29,30]. Unlike prior less-marked heat waves, its health impact attracted considerable public interest and drew attention to the need for efficient alert systems. Moreover, the Intergovernmental Panel on Climate Change predicts an increase in extreme climatic events in the twenty-first century, [31] and several scenario studies, e.g. Beniston's study, [32] predict that heat waves like that in 2003 may occur every two or three years on average, by the third part of this century.

In that context, the objective of this paper is to describe and model the relationship between mortality and temperature in France over a 29-year period (from 1975 to 2003) and, more generally, to propose an approach for the selection of the most predictive combination of temperature factors with a view to predicting the risk of short-term mortality in summer (June to September).

## Methods

The study analysed the relationship between daily fluctuations in mortality and temperature for the whole of France, over the 122 summer days, from 1st June to 30th September of each year, from 1975 to 2003, i.e. 3,538 summer days in all.

### Mortality data

The mortality data were provided by the French National Institute for Medical Research (Inserm). The daily counts of all-cause mortality (*O*_*t*_) for people aged 55 years and over were analysed. The use of this mortality data in the frame of epidemiological studies has been authorised by the French National Commission for Data protection and theLiberties(CNIL).

The yearly population estimates were supplied by the French National Institute of Statistics and Economic Studies (INSEE). Mortality was expressed as the daily mortality rate per 100,000 subjects.

### Climatic data

The daily minimum and maximum temperatures (*Tmin *and *Tmax*) and minimum and maximum relative humidities (*Hmin *and *Hmax*) were recorded by 97 weather stations considered representative of the climate affecting the populations of the 96 French *départements *by the national meteorological service (*Météo-France*). The national daily values of those climatic indicators were the average of those 97 values, weighted by the populations of the *départements*.

A 10-day moving average of the mean temperature (average of the daily minimum and maximum temperatures) was also calculated.

A cumulative temperature variable which was close to the total degree-days of excedance, was constructed [27]. For each day, the cumulative minimum/maximum temperature variable (*CTmin *and *CTmax*) was defined as the sum of the number of degrees above a cut-off point from the current day *t *to either day *t-10 *or the last day with a temperature higher than the cut-off point. This variable was equal to zero if the temperature was below the cut-off point on the day considered:

CTmaxt=∑d=0k(Tmaxt−d−cut-off)×ITmaxt−d>cut-off
 MathType@MTEF@5@5@+=feaafiart1ev1aaatCvAUfKttLearuWrP9MDH5MBPbIqV92AaeXatLxBI9gBaebbnrfifHhDYfgasaacH8akY=wiFfYdH8Gipec8Eeeu0xXdbba9frFj0=OqFfea0dXdd9vqai=hGuQ8kuc9pgc9s8qqaq=dirpe0xb9q8qiLsFr0=vr0=vr0dc8meaabaqaciaacaGaaeqabaqabeGadaaakeaacqWGdbWqcqWGubavieGacqWFTbqBcqWFHbqycqWF4baEdaWgaaWcbaGaemiDaqhabeaakiabg2da9maaqahabaGaeiikaGIaemivaqLae8xBa0Mae8xyaeMae8hEaG3aaSbaaSqaaiabdsha0jabgkHiTiabdsgaKbqabaGccqGHsislcqWGJbWycqWG1bqDcqWG0baDcqqGTaqlcqWGVbWBcqWGMbGzcqWGMbGzcqGGPaqkcqGHxdaTcqWGjbqsdaWgaaWcbaGaemivaqLae8xBa0Mae8xyaeMae8hEaG3aaSbaaWqaaiabdsha0jabgkHiTiabdsgaKbqabaWccqGH+aGpcqWGJbWycqWG1bqDcqWG0baDcqqGTaqlcqWGVbWBcqWGMbGzcqWGMbGzaeqaaaqaaiabdsgaKjabg2da9iabicdaWaqaaiabdUgaRbqdcqGHris5aaaa@684D@

in which: k is the lower of the value 10 and the value of the first previous day on which *Tmax*_*t *_fell below the cut-off point; ITmaxt−d>cut-off
 MathType@MTEF@5@5@+=feaafiart1ev1aaatCvAUfKttLearuWrP9MDH5MBPbIqV92AaeXatLxBI9gBaebbnrfifHhDYfgasaacH8akY=wiFfYdH8Gipec8Eeeu0xXdbba9frFj0=OqFfea0dXdd9vqai=hGuQ8kuc9pgc9s8qqaq=dirpe0xb9q8qiLsFr0=vr0=vr0dc8meaabaqaciaacaGaaeqabaqabeGadaaakeaacqWGjbqsdaWgaaWcbaGaemivaqfcbiGae8xBa0Mae8xyaeMae8hEaG3aaSbaaWqaaiabdsha0jabgkHiTiabdsgaKbqabaWccqGH+aGpcqWGJbWycqWG1bqDcqWG0baDcqqGTaqlcqWGVbWBcqWGMbGzcqWGMbGzaeqaaaaa@415E@ is equal to 1 if Tmax_t-d _is higher than the cut-off and 0 otherwise.

The cut-off points were selected by minimising the deviance of the model including the minimum/maximum temperatures and the minimum/maximum cumulative variables over a grid of cut-off values. The cut-off point for maximum temperatures was found to be equal to 27°C (80.6°F). The cut-off point for minimum temperature was so close to 0°C that the cumulative variable for minimum temperature was very strongly correlated with the moving average of the mean temperature (0.95). Therefore, *CTmin *was not included in the model.

### Statistical analysis

The daily mortality rates were modelled using a generalized estimating equations (GEE) approach, with a Poisson distribution. This model enables both specification of an over-dispersion term and a first-order autoregressive structure that accounts for the autocorrelation of the daily numbers of deaths within each summer and assumes the independence of the summers. A log-linear long-term mortality trend (*Trend*) and the seasonality of mortality during summer, using a quadratic time function by day (*Season*), were included in the model. The model was also adjusted for a dummy variable (*Summer*) which differentiated the 122 summer days (from June to September inclusive) from the other days of the year. The non-summer days provided useful information on the long-term trend of the baseline mortality.

The baseline model *M*_0 _was:

(*M*_0_)   *Log *[*E *(*O*_*t*_)] = *Log *(*PopJ*) + *μ *+ *β Trend *+ *Season*

In which,*PopJ *was the population estimate for the year considered.

The temperature factors were added to the baseline model to yield the model *M*_1_:

(M1)Log[E(Ot)]=Log(PopJ)+μ+β Trend+Season+Summer×[∑iθiTemperature factori,t]
 MathType@MTEF@5@5@+=feaafiart1ev1aaatCvAUfKttLearuWrP9MDH5MBPbIqV92AaeXatLxBI9gBaebbnrfifHhDYfgasaacH8akY=wiFfYdH8Gipec8Eeeu0xXdbba9frFj0=OqFfea0dXdd9vqai=hGuQ8kuc9pgc9s8qqaq=dirpe0xb9q8qiLsFr0=vr0=vr0dc8meaabaqaciaacaGaaeqabaqabeGadaaakeaafaqabeqacaaabaGaeiikaGIaemyta00aaSbaaSqaaiabigdaXaqabaGccqGGPaqkaeaacqWGmbatcqWGVbWBcqWGNbWzcqGGBbWwcqWGfbqrcqGGOaakcqWGpbWtdaWgaaWcbaGaemiDaqhabeaakiabcMcaPiabc2faDjabg2da9iabdYeamjabd+gaVjabdEgaNjabcIcaOiabdcfaqjabd+gaVjabdchaWjabdQeakjabcMcaPiabgUcaRGGaciab=X7aTjabgUcaRiab=j7aIjabbccaGiabdsfaujabdkhaYjabdwgaLjabd6gaUjabdsgaKjabgUcaRiabdofatjabdwgaLjabdggaHjabdohaZjabd+gaVjabd6gaUjabgUcaRiabdofatjabdwha1jabd2gaTjabd2gaTjabdwgaLjabdkhaYjabgEna0oaadmaabaWaaabuaeaacqWF4oqCdaWgaaWcbaGaemyAaKgabeaakiabdsfaujabdwgaLjabd2gaTjabdchaWjabdwgaLjabdkhaYjabdggaHjabdsha0jabdwha1jabdkhaYjabdwgaLjabbccaGiabdAgaMjabdggaHjabdogaJjabdsha0jabd+gaVjabdkhaYnaaBaaaleaacqWGPbqAcqGGSaalcqWG0baDaeqaaaqaaiabdMgaPbqab0GaeyyeIuoaaOGaay5waiaaw2faaaaaaaa@8D6C@

in which the temperature factors are the minimum and maximum temperatures (*Tmin *and *Tmax*), the moving average of the mean temperature (*MA*) and the cumulative variable for maximum temperatures (*CTmax*).

In order to distinguish the specific impact of temperatures up to 5 days before death, the lagged minimum/maximum temperatures and cumulative maximum temperature were also included in the model. Some interactions between minimum/maximum temperatures and the cumulative indicator, recorded on the day considered and the preceding two days, were also added. The full model *M*_1 _thus contained 19 different temperature indicators and 10 interactions (Table 1).

**Table 1:** Description of the 17 groups of temperature variables

**Category**	**Group**		**Variables**	
Group with the moving average of mean temperatures	GMA	MA		

Groups with minimum and maximum temperatures	G1_t_	Tmin_t_	Tmax_t_	Tmin_t _× Tmax_t_
	G1_t-1_	Tmin_t-1_	Tmax_t-1_	Tmin_t-1 _× Tmax_t-1_
	G1_t-2_	Tmin_t-2_	Tmax_t-2_	Tmin_t-2 _× Tmax_t-2_
	G1_t-3_	Tmin_t-3_	Tmax_t-3_	
	G1_t-4_	Tmin_t-4_	Tmax_t-4_	
	G1_t-5_	Tmin_t-5_	Tmax_t-5_	
	G2	Tmin_t_	Tmax_t-1_	Tmin_t _× Tmax_t-1_
	G2'	Tmax_t_	Tmin_t-1_	Tmax_t _× Tmin_t-1_

Groups with maximum temperature and the cumulative variable of maximum temperature	GCum1_t_	Tmax_t_	CTmax_t_	Tmax_t _× CTmax_t_
	GCum1_t-1_	Tmax_t-1_	CTmax_t-1_	Tmax_t-1 _× CTmax_t-1_
	Gcum1_t-2_	Tmax_t-2_	CTmax_t-2_	Tmax_t-2 _× CTmax_t-2_
	GCum1_t-3_	Tmax_t-3_	CTmax_t-3_	
	GCum1_t-4_	Tmax_t-4_	CTmax_t-4_	
	GCum_t-5_	Tmax_t-5_	CTmax_t-5_	
	GCum2	CTmax_t_	Tmax_t-1_	CTmax_t _× Tmax_t-1_
	GCum2'	CTmax_t-1_	Tmax_t_	CTmax_t-1 _× Tmax_t_

*MA*: 10-day moving average of mean temperature.

*Tmin*_*t*-k_: minimum temperature for day t-k; *Tmax*_*t-k*_: maximum temperature for day t-k; k = 0,..., 5

*CTmax*_*t*-k_: cumulative variable of maximum temperature for day t-k;

In a sensitivity analysis, the model was also adjusted for the daily minimum/maximum relative humidities, both as individual factors and as interactions with temperature, as confounder indicators. However, the results did not change.

### Definition of temperature variables

In order to select the most predictive temperature indicators among the 29 variables used in the present paper, a "backward" method was applied on model *M*_1_.

First, the decision was taken to divide the 29 temperature variables and interactions into 17 groups, in order to ensure that the interactions between two indicators were systematically included in the model with the main effects (Table 1).

Most groups contained indicators of the same lag day (G1, GCum1). Four groups contained one temperature indicator recorded on the day considered and another indicator recorded on the preceding day (G2, G2', GCum2 and GCum2').

The 17 groups of indicators were divided in three categories. The first category contained the moving average of the mean temperatures, which reflects the climatic environment in which the subjects lived over the preceding ten days (GMA). The second category contained the minimum and maximum temperatures recorded on various lag days and thus reflected the specific exposure for each day (G1, G2 and G2'). The last category characterised the long periods of high temperatures and therefore included the cumulative indicators (GCum1, GCum2 and GCum2').

### Selection of temperature variables

With a GEE approach, common likelihood-based measures of model fit, like the AIC criterion, cannot be used. Since the objective was to identify the indicators that would provide the best prediction of daily mortality, the criterion chosen for backward elimination of the groups was based on the change in the over-dispersion measured over the period (years) used for estimation.

Over-dispersion was defined as: Φ=[1N−Nvar∑t(Ot−O^t)2O^t]
 MathType@MTEF@5@5@+=feaafiart1ev1aaatCvAUfKttLearuWrP9MDH5MBPbIqV92AaeXatLxBI9gBaebbnrfifHhDYfgasaacH8akY=wiFfYdH8Gipec8Eeeu0xXdbba9frFj0=OqFfea0dXdd9vqai=hGuQ8kuc9pgc9s8qqaq=dirpe0xb9q8qiLsFr0=vr0=vr0dc8meaabaqaciaacaGaaeqabaqabeGadaaakeaacqqHMoGrcqGH9aqpdaWadaqaamaalaaabaGaeGymaedabaGaemOta4KaeyOeI0IaemOta4ecbiGae8NDayNae8xyaeMae8NCaihaamaaqafabaWaaSaaaeaadaqadaqaaiabd+eapnaaBaaaleaacqWG0baDaeqaaOGaeyOeI0Iafm4ta8KbaKaadaWgaaWcbaGaemiDaqhabeaaaOGaayjkaiaawMcaamaaCaaaleqabaGaeGOmaidaaaGcbaGafm4ta8KbaKaadaWgaaWcbaGaemiDaqhabeaaaaaabaGaemiDaqhabeqdcqGHris5aaGccaGLBbGaayzxaaaaaa@4943@

In which: *O*_*t *_was the daily observed counts of all-cause mortality for subjects aged 55 years and over; O^t
 MathType@MTEF@5@5@+=feaafiart1ev1aaatCvAUfKttLearuWrP9MDH5MBPbIqV92AaeXatLxBI9gBaebbnrfifHhDYfgasaacH8akY=wiFfYdH8Gipec8Eeeu0xXdbba9frFj0=OqFfea0dXdd9vqai=hGuQ8kuc9pgc9s8qqaq=dirpe0xb9q8qiLsFr0=vr0=vr0dc8meaabaqaciaacaGaaeqabaqabeGadaaakeaacuWGpbWtgaqcamaaBaaaleaacqWG0baDaeqaaaaa@2F80@ the corresponding estimate; *N *the number of observed days; and *Nvar *the number of variables in the model.

At each step, the "backward" method excluded the group which decreased the over-dispersion least, until all the groups had been excluded from the model. As a sensitivity analysis, the QIC criterion, which is an extension of Akaike's information criterion for GEE models, was also used as a backward elimination criterion, but the results were little changed [33].

### Adequacy and predictive performance of the model

In order to analyse the predictive performance of the model, the 29 years of the period were divided into two distinct groups: a calibration group (e.g., the 28 years from 1975 to 2002), which was used to estimate the parameters and measure the fit of the model estimates with the observations and a validation group (e.g., 2003) which was used for prediction. Using the validation group, comparison of the daily observed and predicted mortality rates enabled assessment of the predictive performance of the model.

In previous studies, a non-linear relationship between minimum/maximum temperatures and mortality has been modelled using spline or smooth functions of same-day temperatures or averaging short-lag values [20,22,23]. A model including natural cubic spline functions with 3 and 6 df for minimum and maximum temperatures for the day *t *and the 2 preceding days was built. The results were compared with those generated with the model obtained by backward selection.

### Sensitivity analyses

The same analysis was conducted on various calibration and validation groups: one calibration group consisted in the 14 even (then odd) years between 1975 and 2002 and the validation group in the 14 odd (then even) years from 1975 to 2002 plus 2003.

In order to measure the sensitivity of the results to the kind of data used, each of the 28 years from 1975 to 2002 was in turn excluded from the calibration group. Conversely, 2003 was also included in the calibration period and the model was estimated using all 29 years (1975–2003).

Lastly, the final model was also assessed separately for men and women, for people aged 55–74 years, for people aged 75 years and over, and for subjects whose causes of death appeared to play a major role in the heat-related excess mortality, i.e., "direct" causes (heatstroke, hyperthermia and dehydration), cardiovascular disease and respiratory disease [7,22,30,34].

## Results

### Descriptive analysis

The average daily mortality rate for subjects aged 55 years and over was 8.5/100,000 person-day for the four summer months (June-September) from 1975 to 2003 (table 2). The highest daily mortality rate was recorded on 12^th ^August 2003 with 20.2 deaths/100,000 person-day.

**Table 2:** Summer (June-September) daily mortality rate, temperature and humidity in France from 1975 to 2003

	**Minimum**	**P10%**	**Mean **(SD)	**P90%**	**Maximum**
**Population aged > 55 years **(million)	11.8	12.0	**15.9 **(1.2)	15.6	16.3

**Daily mortality rate - **per 100,000 subjects/day					
All causes of death, > 55 years	6.28	7.40	**8.48 **(1.02)	9.69	20.15
Three MCD^a^, > 55 years	2.27	2.91	**3.71 **(0.70)	4.61	10.41
All causes of death, 55–74 years	2.58	3.20	**4.03 **(0.75)	5.13	7.80
All causes of death, > 75 years	14.48	17.58	**20.41 **(2.56)	23.25	53.09
Men, > 55 years	7.02	8.77	**10.56 **(1.51)	12.60	18.12
Women, > 55 years	5.41	6.88	**8.24 **(1.22)	9.84	22.51

**Daily climatic indicators**					
Minimum temperature	4.18	9.63	**13.17 **(2.65)	16.54	20.72
Maximum temperature	14.14	18.83	**23.74 **(3.84)	28.89	37.54
Minimum relative humidity	21.33	37.54	**49.32 **(8.98)	60.93	78.09
Maximum relative humidity	77.31	88.21	**92.68 **(3.31)	96.41	99.61

P10% (P90%): 10th (90th) percentile; SD: standard deviation

^a^: Three medical causes of deaths: cardiovascular disease, respiratory disease, and directly heat-related causes (heatstroke, hyperthermia and dehydration)

The mean daily minimum and maximum temperatures in France for the 29 summer periods were 13.2 and 23.7°C, respectively (table 2).

**Figure 1:**
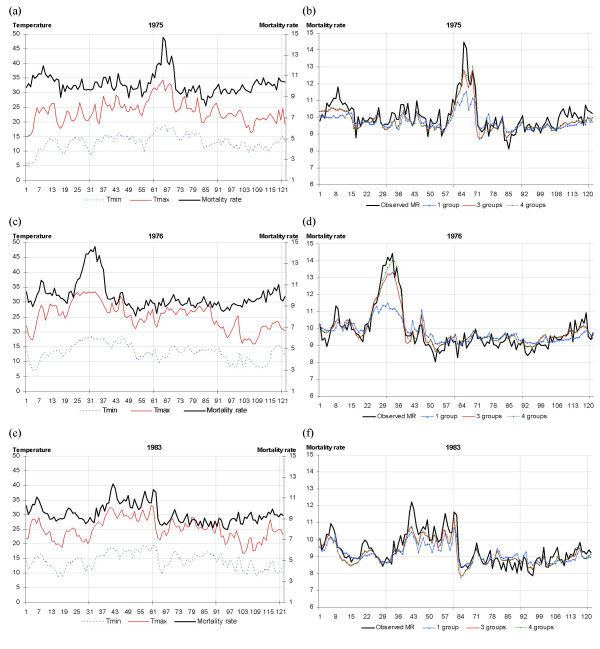
**Fluctuations in daily observed and estimated mortality rates during three summers (1975, 1976, 1983), France**. Fluctuations in daily observed (black) and estimated mortality rates by model with 1 group^a ^(right column, blue), 3 groups^b ^(right column, red) and 4 groups^c ^(right column, green) for minimum (left column, broken blue) and maximum (left column, red) temperatures in France from June through September, 1975, 1976 and 1983. X-axis: days from 1st June to 30th September (122 days). Y-axis: daily mortality rate (deaths/100,000/day). ^a ^1 group: G2; ^b ^3 groups: G2, GMA, GCum1_t_; ^c ^4 groups: G2, GMA, GCum1_t_, Gcum1_t-2_.

As Figure 1 (left column) shows, the daily fluctuations in temperature and mortality rate were closely correlated. Marked peaks in the daily mortality rate occurred, in particular, in the summers of 1975 and 1976. The peaks were concomitant with increasing temperatures and a positive value of the cumulative variable for maximum temperature.

### Selection of the most predictive climatic variables

The model was first estimated over the 28-year period from 1975 to 2002. The over-dispersion of the baseline model *M*_0 _was 5.6, while that of the full model was close to 2.1 (table 3). The lag 1 autocorrelation of the residuals for the baseline model *M*_0 _(0.8) was halved in the full model (0.4).

**Table 3:** Over-dispersion for the calibration period and the validation period, by group of temperature indicators

		**Over-dispersion**	
		
**Group excluded from the full model**	**Number of temperature variables**	**Calibration period 1975–2002**	**Validation period 2003**
**Full model**	29	2.10	3.67
**- G1**_**t-5**_	28	2.10	3.67
**- G1**_**t-1**_	27	2.10	3.67
**- GCUM2'**	26	2.10	3.32
**- G1**_**t**_	25	2.10	3.36
**- GCUM1**_**t-4**_	24	2.10	3.36
**- G2'**	22	2.10	3.33
**- GCUM1**_**t-3**_	21	2.10	3.39
**- GCUM2**	20	2.10	3.34
**- G1**_**t-4**_	18	2.11	3.33
**- G1**_**t-3**_	16	2.11	3.33
**- GCUM1**_**t-5**_	14	2.11	3.51
**- GCUM1**_**t-1**_	12	2.12	4.55
**- G1**_**t-2**_	10	2.13	4.28
**- GCUM1**_**t-2**_	7	2.22	13.61
**- GCUM1**_**t**_	4	2.73	67.49
**- GMA**	3	3.14	59.97

**- G2 (= model M_**0**_)**	**0**	**5.64**	**133.71**

The groups of temperature indicators were selected using the backward method.

With regard to the change in overall over-dispersion in the backward elimination of temperature factor groups, the over-dispersion remained quite steady from the full model to that only containing the following four groups (table 3):

- G2: the minimum temperature for a day *t*, the maximum temperature on the preceding day and their interaction,

- GMA: the 10-day moving average of mean temperature,

- GCum1_t_: the maximum temperature for a day *t*, the cumulative variable for maximum temperatures for a day *t *and their interaction,

- GCum1_t-2_: the maximum temperature 2 days before death, the cumulative variable for maximum temperatures 2 days before death and their interaction.

Subsequently, the over-dispersion rose sharply until all the groups had been excluded from the model.

The interactions turned out to contribute strongly to explaining the variation in daily mortality (table 4), although caution is required in the interpretation of the estimated parameter coefficients, since there are correlations between covariates.

**Table 4:** Covariate estimates for the model with four groups (G2, GMA, GCum1_t_, Gcum1_t-2_)

**Temperature indicator**	**Coefficient estimate β (SE)**	**Percentile P10% – P90% of the indicator**	**Relative risk estimate of P90%/P10%**^**a**^	**Pr > |Z|**^b^
**G2**				
Tmin_t_	-0.0219 (0.0027)	8.74 – 17.35	0.83	0.0145
Tmax_t-1_	-0.0124 (0.0018)	17.74 – 29.98	0.86	0.2561
Tmin_t _× Tmax_t-1_	0.0013 (0.0001)	164.25 – 515.27	1.58	0.0000

**GMA**				
Moving average	-0.0158 (0.0009)	14.60 – 22.52	0.88	0.0000

**GCum1**_**t**_				
Tmax_t_	0.0045 (0.0006)	17.75 – 29.98	1.06	0.0000
Ctmax_t_	-0.007 (0.0026)	0.00 – 10.17	0.93	0.2024
Tmax_t _× Ctmax_t_	0.0003 (0.0001)	0.00 – 298.61	1.09	0.0015

**GCum1**_**t-2**_				
Tmax_t-2_	-0.0002 (0.0006)	17.75 – 29.98	1.00	0.9319
Ctmax_t-2_	-0.0093 (0.0027)	0.00 – 10.17	0.91	0.0181
Tmax_-2 _× Ctmax_t-2_	0.0004 (0.0001)	0.00 – 298.61	1.13	0.0000

^a ^Multiplying factor for the increase in mortality when the indicator increases from the 10^th ^percentile (P10%) to the 90^th ^percentile (P90%),

^b ^Pr > |Z| is the significance probability

### Adequacy of the model

Figure 1 (right column) shows the daily fluctuations in observed and estimated mortality rates for three summers (1975, 1976 and 1983) by the number of groups included in the model. Those summers were marked by excess mortality rates related to periods of extreme temperatures, which differed in terms of their intensities and temporal configurations.

For days with usual temperatures, the estimates of the daily mortality rate generated by the model only incorporating the group of temperature factors G2 (minimum and maximum temperatures for a day *t *and preceding day and their interaction) were neither improved nor impaired by the inclusion of the other groups (GMA and GCum1_t_) (Figure 1).

In contrast, group G2 was not sufficient to estimate the daily mortality rates during extreme events and the inclusion of group GCum1_t _in the model greatly improved the estimates. This finding was particularly marked for the heat episodes in 1975 and 1976 (Figure 1). The fourth group selected by the backward method (GCum1_t-2_) improved the mortality estimates for the 1976 heat waves only.

For the whole 28-year period (1975–2002), the correlation between the observed and the estimated mortality ratios was 0.88 with the model with four groups.

### Predictive performance of the model

**Figure 2:**
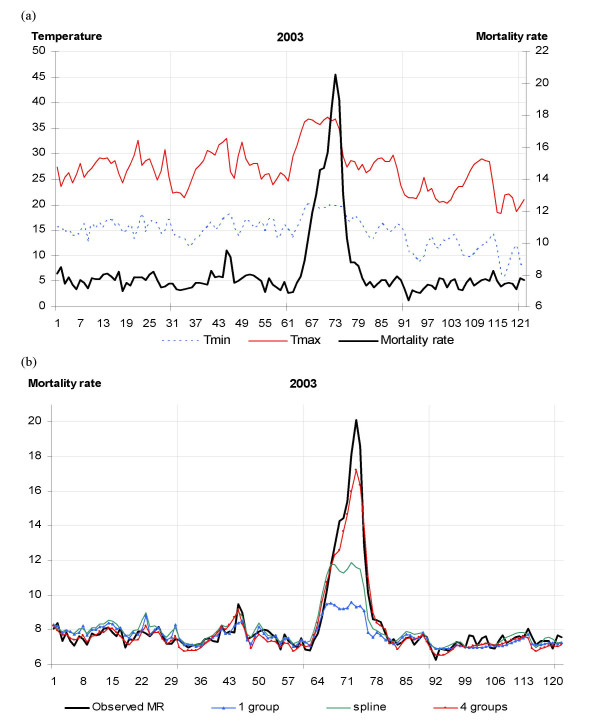
**Fluctuations in daily observed and predicted mortality rates, in France in the summer of 2003**. (a) Fluctuation in daily minimum (broken blue) and maximum (red) temperatures, and observed mortality rates (black) in France from 1^st ^June to 30^th ^September, 2003. (b) Fluctuations in daily observed (black) and predicted mortality rates, by model with 1 group^a ^(blue) and 4 groups^b ^(red), and by model with cubic spline functions for minimum and maximum temperatures ^c ^(green), in France from 1^st ^June to 30^th ^September, 2003. X-axis: days from 1st June to 30th September (122 days). Y-axis: daily mortality rate (deaths/100,000/day). ^a ^1 group: G2; ^b ^4 groups: G2, GMA, GCum1_t_, Gcum1_t-2_; ^c ^model with natural cubic spline functions (6 df) for minimum and maximum temperatures of same-day and 2 lag days.

For the validation year 2003, the model with 1 group provided a satisfactorily prediction of the daily number of deaths, compared to the observed deaths, for the days with usual temperatures (Figure 2b). The prediction for the 2003 heat wave (from 1^st ^to 20^th ^August) was greatly improved when the third and fourth groups were added (Figure 2b).

The model with four groups explained 97% of the extra-Poisson variability of the daily mortality rates observed during summer 2003 (Table 3).

For the days with usual temperatures, the mortality estimates obtained with the model including cubic spline functions were close to those obtained with the model with four groups (including minimum/maximum temperatures, the cumulative indicator of maximum temperatures and their interactions). However, the model with four groups provided much better estimates of the daily mortality rates during the 2003 heat wave than the model with splines (Figure 2b).

Sensitivity analysis

The sensitivity of the approach was first assessed by evaluating the change in the results when each of the 28 years was excluded in turn from the calibration group or by using calibration groups consisting of either the 14 even or 14 odd years from 1975 to 2002 (table 5). For the backward method, the three most predictive temperature groups were similar to those selected in the main analysis, irrespective of the year excluded from the calibration group. However, the regression coefficient of the temperature parameters was subject to change. In particular, the coefficient of the cumulative variable for maximum temperature was weaker when a year including an extreme climatic event, such as 1975 or 1976, was excluded.

**Table 5:** Over-dispersion by population subgroup and temperature group

			**Full Model**	**5 groups**	**4 groups**	**3 groups**	**2 groups**	**1 group**	**Model M**_**0**_
**Population subgroup**	**55–74 years**	Group		**G1**_**t-2**_	**GCum1**_**t-2**_	**G2**	**GMA**	**GCum1**_**t**_	
		Over-dispersion	1.80	1.80	1.80	1.82	1.90	1.97	**2.49**
	
	**75 years and more**	Group		**GCum1**_**t-4**_	**G1**_**t-2**_	**GCum1**_**t**_	**GMA**	**G2**	
		Over-dispersion	2.09	2.11	2.13	2.21	2.61	2.90	**5.26**
	
	**Men**	Group		**GCum1**_**t-1**_	**G1**_**t-2**_	**GCum1**_**t**_	**GMA**	**G2**	
		Over-dispersion	1.46	1.47	1.47	1.49	1.66	1.84	**2.61**
	
	**Women**	Group		**G1**_**t-2**_	**GCum1**_**t-2**_	**GMA**	**GCum1**_**t**_	**G2**	
		Over-dispersion	1.71	1.71	1.72	1.79	2.11	2.37	**4.22**

**Three medical causes of death**^a^		Group		**GCum1**_**t-1**_	**G1**_**t-2**_	**GMA**	**GCum1**_**t**_	**G2**	
	Over-dispersion	1.74	1.76	1.79	1.84	2.10	2.33	**3.81**	

**Calibration group odd/even years subgroup**	**14 even years**	Group		**G1**_**t-2**_	**GCum1**_**t-2**_	**G2**	**GMA**	**GCum2'**	
		Over-dispersion	2.06	2.06	2.07	2.11	2.61	2.98	**5.66**
	
	**14 odd years**	Group		**GCum1**_**t-1**_	**G1**_**t-2**_	**GCum1**_**t**_	**GMA**	**G2**	
		Over-dispersion	2.16	2.19	2.23	2.29	2.70	3.15	**5.60**

^a^: Three Medical Causes of Death: heat-related deaths (hyperthermia, heatstroke, dehydration), cardiovascular disease and respiratory disease

The 5 groups of temperature indicators were the last 5 groups excluded from the model using the backward method.

When summer 2003 was included in the calibration period, only the estimates of the cumulative indicator parameters were higher. The daily mortality rate estimates for the 1976 and 2003 heat waves were improved but the estimates for the other days were unchanged.

Lastly, the same analysis was conducted separately for men and women, for subjects aged 55–74 years, and for subjects aged 75 years and over, for the three main medical categories of causes of heat-related excess mortality (cardiovascular disease, respiratory disease and directly heat-related deaths). The three groups, G2, GMA and GCum1_t_, were again the most predictive of the daily fluctuations in mortality since 1975 (table 5).

## Discussion

This paper describes the relationship between the daily fluctuations in mortality and temperatures over a 29-year period (1975–2003) in France as a whole. It also proposes and validates an approach to determining the optimum combination of temperature indicators to explain both the usual daily fluctuations in mortality and the excess mortality associated with intense summer heat episodes.

Although temperatures are heterogeneous in different places in France, the daily population-weighted average of temperatures on a national scale turned out to be highly correlated with the daily number of deaths in summer.

The results are consistent with previous studies [15,16,19,21-23,28] and provide additional quantitative information on the summer temperature-mortality relationship.

The major part of the daily fluctuations in summer mortality is explained by the minimum and maximum temperatures observed for a day *t *and the preceding days and their interaction. Both minimum and maximum high temperatures have been shown to have a significant impact on mortality in summer [2,7,8]. Cool summer nights have been reported to allow recuperation when daytime temperatures are high.

However, the daily absolute temperatures do not appear sufficient to explain both the daily fluctuations in the usual mortality rates and the excess mortality rates related to extreme events. The interaction between the cumulative effect of temperatures above a cut-off point over a period of consecutive days and the maximum temperature appeared more predictive of the mortality during heat episodes.

Three recent studies have drawn attention to the importance of using a cumulative indicator of hot days or degrees above a cut-off to measure the magnitude of a heat wave in terms of its intensity and duration [7,27,28]. While the cumulative indicator has rarely been studied, it may be of value in predicting mortality during heat waves.

The cumulative indicator depends on the choice of the cut-off point. In this paper, the cut-off (27°C) was determined by considering the national values for daily maximum temperatures and should not be interpreted as equivalent to the cut-off in a similar analysis of a single city. If the temperature on a given day in France is, say, 25°C, then many localities will obviously have temperatures above 27°C. Moreover, the cut-off point depends on the population considered. If the cumulative indicator is to be used for another population, the cut-off needs to be adapted to the data, in order to take into account the population's specificities.

It is also important to note that the long time period considered herein contained several heat episodes (particularly in 1975 and 1976), which differed in terms of intensity, duration, temporal configuration and geographic extent. Extreme events in the calibration period are necessary in order to enable satisfactory estimation of the cumulative temperature parameter.

This study was designed to enable fine analysis of the role of temperature in the fluctuations of the daily mortality rate. From the 29 temperature indicators and interactions used in the present study, 17 groups were formed and 10 variables were finally selected as the most predictive indicators. The fact that alternative combinations of indicators in groups, possibly including other climatic indicators, might yield an equally good or better predictive performance with respect to daily mortality cannot be ruled out. However, the selected temperature indicators explained 76% of the total extra-Poisson variability, demonstrating the great importance of the selected indicators with respect to summer mortality.

The present model contains a cumulative indicator and several interactions, which are non-linear functions of temperatures. The results show that the interactions are the most predictive indicators of the daily fluctuations in mortality. A comparative analysis, using the model including cubic spline functions of same-day temperatures and short-lag values has also shown that the model with four groups provided much better estimates of the daily mortality rates during the heat episodes, in particular for the summers of 1976 and 2003.

Even though temperature has been shown to be the main indicator of mortality, other environmental factors may also influence the fluctuations in daily mortality.

Humidity has often been studied, either as an individual factor or in the form of an index combining temperature and humidity, such as the apparent temperature or discomfort index [15,16,18,19,22,24,25]. The results of those studies were not consistent and depended on the usual climatic characteristics of the countries in which the studies were conducted. Wind speed and pressure have also been studied, but have rarely been found to be significantly associated with mortality. In the present study, humidity did not improve the daily mortality estimates. This finding may reflect the fact that, in France, from 1975 to 2003, the particularly hot days were not highly humid.

Air pollution has also been reported to have an effect on mortality during extreme climatic events, particularly in urban areas [18,20,22,23,25]. To the authors' knowledge, the relationship between temperature, air pollution and mortality has not been studied on the wide geographic scale of a whole country. The present model did not include air pollution indicators, since country-wide data on air pollution for the 29-year period were not available. However, an analysis with fine pollution and temperature adjustment might enable estimation and dissociation of the respective roles of air pollution and temperature with regard to daily mortality in summer and might explain a part of the 24% unexplained extra-Poisson variability.

Few studies have studied the weather-mortality relationship on a wide geographic scale [8,21,27]. Most of the previous studies have addressed the relationship with respect to one or several large urban areas. Such areas may have more homogeneous environmental, epidemiologic and demographic characteristics. In contrast, the present study analysed the mortality of the French population aged 55 years and over, equivalent to an average daily death count of 1100 (standard deviation: 110). So doing markedly reduced the variability related to small frequencies in studies of specific urban areas. However, the mortality and climate of the various regions of France are heterogeneous. A finer geographic analysis of the mortality-temperature relationship might therefore improve both the overall and local predictive performance with respect to mortality.

This paper is based on the data recorded over the 28-year period prior to 2003. In France, prior to 2003, summer heat was not considered to constitute a major death risk and only limited national preventive and warning systems existed. Thus, the mortality-temperature relationship analysed herein was not influenced by the effects of intensive measures to reduce heat-related mortality.

Since the dramatic European heat wave in summer 2003, the awareness of the risk associated with summer heat, behavioural adaptation to high temperatures during extremely hot weather and the set-up of an alert system have probably modified the mortality-temperature relationship. A national Heat Health Watch Warning System has been created to prevent the mortality associated with extreme heat episodes. The system is operational every year from 1^st ^June to 1^st ^September on the national scale [35].

Thus, the model presented herein may be pertinent with respect to evaluating and, possibly, refining the existing warning system by providing a quantitative dimension to the prediction of the mortality risk on a wide geographic scale. The quantitative estimate could then be used by the health authorities to evaluate the magnitude of the impact in terms of short-term mortality when a heat wave is predicted by the meteorological services. The estimate would also enable set up of an emergency plan and operations that would be commensurate with the severity of the heat episode.

## Conclusion

Although France is a large country with marked geographic heterogeneity both in mortality and temperatures, a strong correlation between the daily fluctuations in mortality and the fluctuations in temperatures in summer was observed on a national scale, over a 29-year period (1975–2003).

A combination consisting in the minimum/maximum temperatures and the cumulative indicator of maximum temperature recorded over short-lag days as well as their interactions was obtained using a backward method. The combination explained 76% of the total extra-Poisson variability of the mortality. The model provided a satisfactory quantitative estimation of the daily mortality both for the days with usual temperatures in summer (June to September) and for days during intense heat episodes.

The results may further contribute to the heat warning system by providing quantitative prediction on the short-term mortality to be expected, on the basis of temperatures, if a heat episode occurs.

## Competing interests

The author(s) declare that they have no competing interests.

## Authors' contributions

DH and EJ were the principal investigators of the study. DH provided statistical and epidemiological expertise and participated in the interpretation of data. EJ contributed to the acquisition of the data on all-cause mortality and mortality by medical cause of death, and participated in the interpretation of data. PF and PB made substantial contributions to the acquisition of the climatic data. AF was in charge of the statistical modelling and analysis of data, and participated in the interpretation of data and drafted the manuscript. GR participated in the statistical analysis and in the interpretation of the data. All the authors revised the manuscript and have approved the final version.

## Pre-publication history

The pre-publication history for this paper can be accessed here:


